# A Polyubiquitin Chain Reaction: Parkin Recruitment to Damaged Mitochondria

**DOI:** 10.1371/journal.pgen.1004952

**Published:** 2015-01-22

**Authors:** Brigit E. Riley, James A. Olzmann

**Affiliations:** 1 Sangamo BioSciences Inc., Richmond, California, United States of America; 2 Department of Nutritional Sciences and Toxicology, University of California, Berkeley, Berkeley, California, United States of America; Stanford University School of Medicine, UNITED STATES

Mutations in the E3 ubiquitin ligase Parkin or the mitochondrial kinase PINK1 cause autosomal recessive forms of Parkinson’s disease [1, 2]. Genetic and cell biological studies have implicated PINK1 and Parkin as critical elements in mitophagy, a mitochondrial quality control pathway that involves the ubiquitin-proteasome system (UPS) and the autophagy-lysosomal system [1, 2]. Under basal conditions, PINK1 is processed by mitochondrial proteases and targeted for degradation by the UPS [1, 2]. Following persistent mitochondrial damage (e.g., treatment with the mitochondrial uncoupling agent CCCP) PINK1 is stabilized and accumulates in an active form on the outer mitochondrial membrane [1, 2]. Although PINK1 activity is essential for the mitochondrial recruitment of cytoplasmic Parkin and for the subsequent ubiquitin-dependent clearance of damaged mitochondria, the mode of Parkin activation and recruitment has been elusive [1, 2].

**Figure 1:**
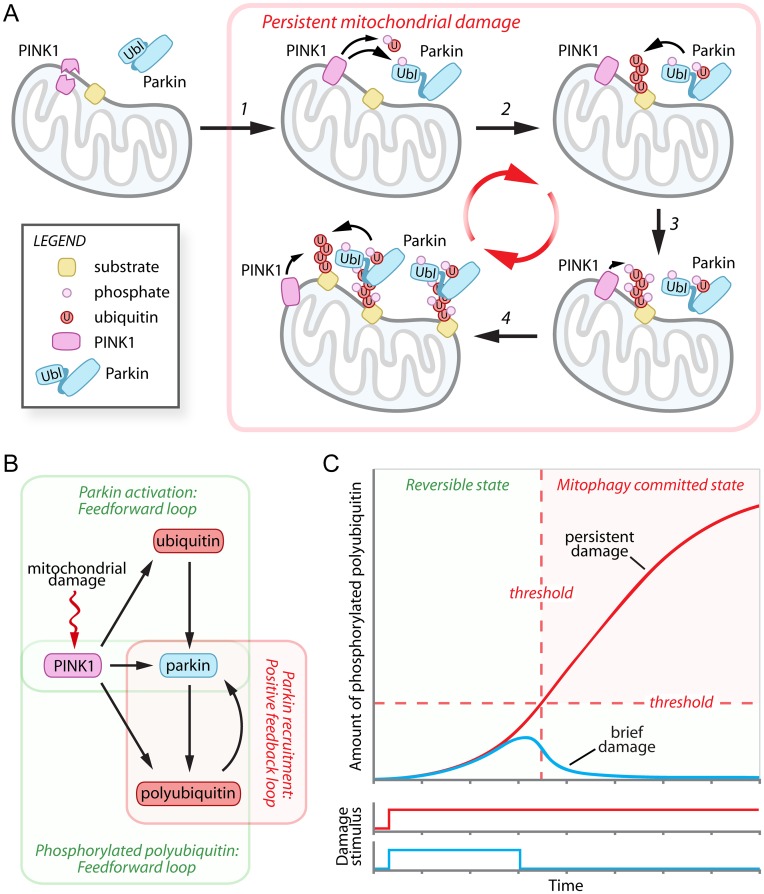
Damage-induced feedforward and positive feedback loops mediate the cellular decision to destroy mitochondria. (A) In healthy cells the mitochondrial kinase PINK1 is proteolytically processed and degraded by the UPS. Following mitochondrial damage, PINK1 is stabilized and phosphorylates ubiquitin and the Parkin UbL (*step 1*), activating Parkin and promoting the polyubiquitination of mitochondrial outer membrane substrates (*step 2*). The polyubiquitin chains are then phosphorylated by PINK1 (*step 3*) and mediate the recruitment of additional Parkin to the mitochondria (*step 4*). (B) Network motifs in the PINK1-Parkin pathway. Mitochondrial damage inhibits PINK1 degradation, initiating dual coherent feedforward loops in the activation of Parkin and generation of phosphorylated polyubiquitin and a positive feedback loop in Parkin recruitment. (C) Hypothetical graph modeling the relationship between mitochondrial damage signal persistence (brief—*blue line*, and persistent—*red line*) and the accumulation of phosphorylated polyubiquitin chains during the progression towards mitophagy commitment. UPS, ubiquitin-proteasome system.

A series of recent papers indicates that PINK1 initiates mitophagy by a two-pronged mechanism involving direct phosphorylation of ubiquitin at serine 65 [3–5] and the ubiquitin-like domain (UbL) of Parkin, also, at serine 65 [6–8] (Fig. 1A, *steps 1–2*). Biochemical and structural analyses of Parkin demonstrated that the unique Parkin domain (UPD):Rcat interface (previously termed RING0:RING2), the repressor element of Parkin (REP):RING1 interface, and potentially the UbL:RING1 interface mediate autoinhibition of Parkin under steady state conditions in the cell [9–14]. It is tempting to speculate that PINK1 phosphorylation of the Parkin UbL and/or the binding of phosphorylated ubiquitin releases the autoinhibitory elements to allow E2~Ub binding or facilitates conformational rearrangements to confer E2~Ub discharge, ultimately leading to exposure of an optimally aligned Parkin active site. Although the studies on PINK1 phosphorylation of ubiquitin [3–5] and Parkin [6–8] suggest a novel mechanism for PINK1 activation of Parkin, the expression of phosphomimetics of ubiquitin and Parkin was insufficient to promote the mitochondrial recruitment of Parkin [3, 8]. Thus the mechanism underlying PINK1-mediated Parkin recruitment remained a mystery.

In December’s issue of *PLOS Genetics*, Shiba-Fukushima et al. provide compelling data indicating that PINK1 directly phosphorylates polyubiquitin chains to mediate the mitochondrial recruitment and activation of Parkin (Fig. 1A, *steps 3–4*) [15]. In vitro, affinity purified Parkin from cells bound purified polyubiquitin chains phosphorylated by recombinant PINK1 with a preference for long K63-linked polyubiquitin over K48-linked polyubiquitin chains, although this preference was not recapitulated with Parkin purified from bacteria. To simulate mitochondrial linked polyubiquitin chains, the authors expressed four tandem copies of ubiquitin G76V fused to the mitochondrial targeting sequence of Tom70 [15]. Phosphomimetic (S65E) versions of the tandem polyubiquitin chains were bound by Parkin through its RING1-BRcat domains (previously termed RING1:IBR) and were able to promote stable mitochondrial association of Parkin even in the absence of mitochondrial damage [15]. Both cytosolic phosphomimetic ubiquitin and mitochondrially targeted phosphomimetic tandem polyubiquitin chains were sufficient to activate Parkin, as evidenced by increased Parkin C431S-ubiquitin oxyester formation, but only when the Parkin UbL phosphomimetic was used [15]. To further understand the physiological importance of their findings, Shiba-Fukushima et al. employed PINK1-/- and Parkin -/- *Drosophila* models, which are associated with severe mitochondrial swelling and matrix disorganization and age-dependent motor defects. Strikingly, the expression of mitochondrially targeted phosphomimetic tandem polyubiquitin chains significantly improve both mitochondrial morphology and motor function in PINK1-/- flies, with little effect on mitochondrial morphology in Parkin -/- flies [15]. These results are in excellent agreement with a recent publication that employed quantitative proteomics to study PINK1-stimulated Parkin polyubiquitination [16]. Together, these two publications [15, 16] suggest that PINK1 phosphorylation of polyubiquitin is the rate-limiting event required for the mitochondrial recruitment, and potentially also the activation, of Parkin.

The destruction of mitochondria represents an irreversible cellular decision with significant consequences for cellular physiology. The emerging data support a model in which the decision to degrade damaged mitochondria is controlled by dual coherent feedforward loops that precede a positive feedback loop (Fig. 1B). In a feedfoward loop, two input factors, one of which controls the other, jointly regulate a third target factor. In the PINK1-Parkin pathway, the first feedforward loop mediates maximal activation of Parkin by PINK1 phosphorylation of the Parkin UbL and of ubiquitin. The second feedforward loop involves the generation of mitochondrial polyubiquitin chains by PINK1-activated Parkin, and/or another mitochondrial E3 ligase, and their subsequent phosphorylation by PINK1. These phosphorylated polyubiquitin chains appear to be capable of initiating a self-propagating positive feedback loop, recruiting Parkin to the mitochondria and presumably stimulating polyubiquitination of mitochondrial substrates, which can then be phosphorylated by PINK1 and recruit additional Parkin. The organization of these network motifs predicts beneficial features with respect to the decision to degrade mitochondria, including an initial delay period and a mechanism to detect the persistence of mitochondrial damage (Fig. 1C). During the delay period (i.e., low levels of phosphorylated polyubiquitin), the decision to commit to mitophagy would be rapidly reversible, providing a useful means of filtering out brief, low levels of mitochondrial damage signals and preventing unwarranted mitochondrial destruction (Fig. 1C, *blue line*). Only a persistent damage stimulus that overcomes a specific threshold would be sufficient to initiate the positive feedback loop and commit mitochondria for mitophagy (Fig. 1C, *red line*). The actions of putative unidentified ubiquitin and Parkin UbL phosphatases, or of mitochondrial deubiquitinating enzymes USP30 [17], USP15 [18], or USP8 [19], which antagonize Parkin-mediated polyubiquitination, would be predicted to regulate the extent of the delay period and the precise commitment threshold. Interestingly, USP30 has been reported to be targeted for UPS degradation by Parkin [17], providing an elegant mechanism to gradually reduce the magnitude of UPS30’s influence during persistent mitochondrial damage.

The new study from Shiba-Fukushima et al. [15] contributes an intriguing model for the role of PINK1 in Parkin recruitment to damaged mitochondria and raises several interesting questions for future investigation. Is an unidentified Parkin UbL and/or ubiquitin phosphatase involved in mitochondrial quality control? The mitochondrial phosphatase PGAM5, which functions downstream of PINK1 [20], is a logical candidate. If polyubiquitination induces the proteasomal degradation of tagged substrates, how does the phospho-polyubiquitin mitochondrial signal persist, are they shielded by phospho-polyubiquitin binding domain-containing proteins or not efficiently recognized by the proteasome? Does Parkin self-association [21] amplify the feedforward loop? How is the binding and exchange of substrates, phospho-polyubiquitin chains and/or phospho-ubiquitin by Parkin coordinated to control the timing of substrate degradation? Finally, it will be imperative to determine the therapeutic potential of small molecule mimetics of phosphorylated ubiquitin (or Parkin UbL) and regulators of the PINK1-Parkin mitochondrial quality control pathway in the search for cures of idiopathic Parkinson’s disease.
